# Modeling Treatment Strategies to Inform Yaws Eradication

**DOI:** 10.3201/eid2611.191491

**Published:** 2020-11

**Authors:** Alex Holmes, Michael J. Tildesley, Anthony W. Solomon, David C.W. Mabey, Oliver Sokana, Michael Marks, Louise Dyson

**Affiliations:** Mathematics Institute, University of Warwick, Coventry, UK (A. Holmes, M.J. Tildesley, L. Dyson);; University of Warwick School of Life Sciences, Coventry (M.J. Tildesley, L. Dyson);; Hospital for Tropical Diseases, London, UK (A.W. Solomon, D.C.W. Mabey, M. Marks);; London School of Hygiene and Tropical Medicine, London (A.W. Solomon, D.C.W. Mabey, M. Marks);; Ministry of Health and Medical Services, Honiara, Solomon Islands (O. Sokana)

**Keywords:** yaws, mass drug administration, Treponema pallidum pertenue, contact tracing, bacteria, bacterial infections, neglected tropical diseases, modeling, Solomon Islands, Treponema pallidum, TPE

## Abstract

Yaws is a neglected tropical disease targeted for eradication by 2030. To achieve eradication, finding and treating asymptomatic infections as well as clinical cases is crucial. The proposed plan, the Morges strategy, involves rounds of total community treatment (i.e., treating the whole population) and total targeted treatment (TTT) (i.e., treating clinical cases and contacts). However, modeling and empirical work suggests asymptomatic infections often are not found in the same households as clinical cases, reducing the utility of household-based contact tracing for a TTT strategy. We use a model fitted to data from the Solomon Islands to predict the likelihood of elimination of transmission under different intervention schemes and levels of systematic nontreatment resulting from the intervention. Our results indicate that implementing additional treatment rounds through total community treatment is more effective than conducting additional rounds of treatment of at-risk persons through TTT.

Yaws is an infectious disease found in South America, Asia, Africa, and Oceania. It is caused by *Treponema pallidum* subspecies *pertenue* (*1*), an organism morphologically identical to *T. pallidum* subsp. *pallidum*, which causes syphilis. Yaws can manifest as skin lesions, involvement of the bones and joints, and eventually irreversible disfigurement. It is spread by direct contact between a susceptible person and lesions of infectious persons and particularly affects persons 2–15 years of age.

In the 1950s, the World Health Organization (WHO) and UNICEF led efforts to eradicate yaws through mass treatment with benzathine benzylpenicillin (*2*), reducing the number of cases worldwide by »95% (*3*). Yaws then fell off the public health agenda and has since resurged in several countries. Eradication efforts were renewed when, in 2012, a study (*4*) showed that treatment with a single oral dose of azithromycin was noninferior to benzathine benzylpenicillin and did not require cold chains, injection equipment, or special training to administer, thus reducing the logistic barriers to mass drug administration and potentially making eradication more feasible.

In 2012, in response to this finding, member states of WHO committed to eradicate yaws by 2020 (*5*), although more recently 2030 has been suggested as a more realistic target (*6*). The primary reason for this change was the high number of countries in which yaws is still endemic, and the even higher number of previously yaws-endemic countries whose current endemicity status is currently unknown. The current eradication strategy, known as the Morges strategy, consists of treatment with single-dose oral azithromycin in 2 modes of community-based intervention: total community treatment (TCT) and total targeted treatment (TTT) (*7*). TCT attempts to treat everyone in a given community (village or town) regardless of the number of active clinical cases, whereas TTT treats active clinical case-patients and their contacts, where contacts are those in the same household or school or are playmates of affected persons (*8*). In response to evidence from pilot studies that a single round of TCT is not sufficient to interrupt transmission, WHO has proposed revising the strategy (*9*). The revised strategy suggests that, in most circumstances, 2–3 rounds of TCT are likely to be required, followed by TTT performed at intervals of 6–12 months (*9*). TCT is designed for situations in which a large proportion of the population is infected, whereas TTT is intended to treat a small number of remaining cases once elimination of transmission (EOT) appears close.

*T. pallidum* subsp. *pertenue* infection can be divided into active yaws and latent yaws. Active yaws can then be split further into primary, secondary, and tertiary yaws (*10*). After an incubation period averaging 21 days (range 9–90 days), primary yaws initially manifests as a papule at the site of inoculation. The papule then enlarges, lasting for 3–6 months. Early secondary yaws lesions might appear near the initial lesion and persist >6 months. These lesions heal spontaneously, leading to a noninfectious latent period that can last the remaining lifetime of the person (*11*). However, the state of latency can end at any time by the reappearance of infectious lesions. Tertiary yaws lesions are now rarely seen (*12*), but when they do manifest, they appear years after primary yaws and are often destructive but are noninfectious.

A considerable challenge for eradicating yaws is the existence of asymptomatic persons, who together harbor a large reservoir of infection. For each active case of yaws, as many as 6–10 cases of latent yaws might exist (*13*). If we are not treating the whole community, successfully treating clinical case-patients and latently infected persons is essential. The assumption conceptually underlying TTT is that asymptomatic persons are likely to be close contacts of existing clinical case-patients.

The difficulty of diagnosing latent yaws in adults also represents a challenge for researchers attempting to understand the dynamics of transmission. Serologic testing cannot distinguish between syphilis and yaws infections; thus, only children <15 years of age typically have serologic tests performed (*3*).

In this article, we extend previous yaws modeling work by incorporating household structure and simulations of eradication strategies into the model. We evaluate the Morges strategy and variants of it for their suitability in meeting the WHO goal of yaws eradication. We investigate the likely effect of different assumptions regarding coverage during rounds of TCT on the success of a strategy and the effect systematic nonadherence could have on its effectiveness. We also consider whether regular surveillance could be an effective component of a program seeking to meet the WHO goal.

## Methods

**Figure 1:**
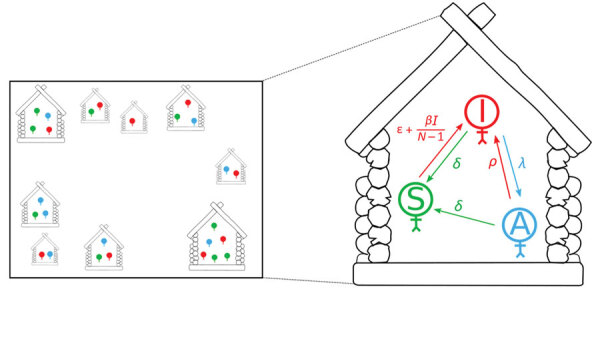
Graphic representation of the model being used for yaws transmission. Each house shape indicates a household, and the number of shapes inside each indicates number of persons. Each person is either susceptible (S, green), infectious (I, red), or asymptomatic (A, blue). Close-up image at right shows details of model parameters (see Tables 1, 2).

We adapted the Markov model developed by Dyson et al. (*14*). The model consists of houses, each containing several inhabitants. Persons might be classified as susceptible, infected and infectious, or asymptomatically infected but not infectious. Within each household, susceptible persons might become infected by other persons from within their household or from other households. From the infectious state, a person can either recover and reenter the susceptible state, or they can have onset of a latent infection, entering the asymptomatic state. From the asymptomatic state, persons can either recover, entering the susceptible state, or the infectious lesions can recur, causing them to reenter the infectious state. These transitions are summarized in Table 1 and Figure 1. In Dyson et al. (*14*) this model was fitted at steady state to data from the Solomon Islands (*13*), using a presumed constant rate of between-household infection. In our study, we extended the model to include a dynamic rate of between-household infection, which we assumed to be proportional to the total prevalence of infectious persons in the population at a given time (Appendix). We took parameter values from posterior distributions with maximum posterior value drawn from expert opinion and previous model-fitting to Solomon Islands data (*14*) (Table 2).

**Table 1:** Permitted state transitions and state transition rates for steady state household model of yaws transmission*

Description	State transition	Rate
Infection, external (ε) and within-household (β)	(S,I,A) ® (S-1,I+1,A)	
Treatment/birth-death	(S,I,A) ® (S+1,I-1,A)	δ
Remission	(S,I,A) ® (S,I-1,A+1)	λ
Recurrence	(S,I,A) ® (S,I+1,A-1)	ρ
Treatment/birth-death	(S,I,A) ® (S+1,I,A-1)	δ

*Methods described in Appendix . A, asymptomatically infected (but not infectious); I, infected and infectious; S, susceptible.

**Table 2:** Parameter values for household structured yaws transmission model, based on data collected from the Solomon Islands*

Parameter	Value	Source
β	0.0516	(*13*)
δ	0.0513	(*13*)
λ	0.185	(*14*,*15*)
ρ	0.0165	(*13*)
ε†	0.004	(*13*)
α‡	0.1669	Appendix

*Given parameter value is the maximum posterior value of the distribution from which each parameter is drawn, with the exception of λ which is consistent with expert opinion. Methods described in Appendix. †Steady state external force of infection. ‡Between-household rate of infection corresponding to a steady state external force of infection ε.

**Figure 2:**
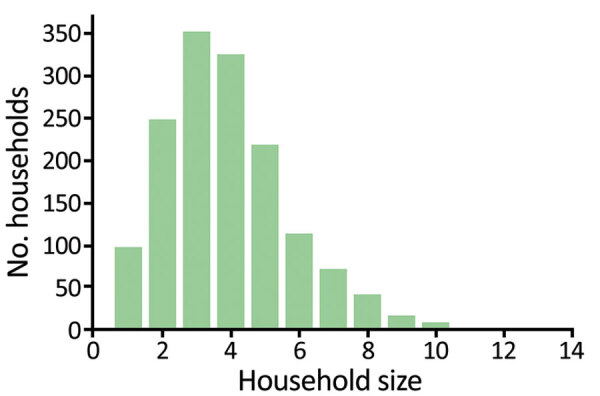
Distribution of household sizes in the model being used for yaws transmission, based on data collected from the Solomon Islands in 2013.

We considered a population of 5,000 households, with sizes distributed according to empirical data from the Solomon Islands (Figure 2), representing a population of »20,000 persons. Each treatment scheme consisted of several rounds of TCT, followed by several rounds of TTT. During TTT, we defined a contact to be anyone in the same household as an infectious person. We assumed an optimistic coverage of 100% of active case-patients and their contacts given treatment during rounds of TTT (a conservative assumption under the hypothesis that TTT is not an effective strategy), TCT coverage of 80% (*3*), and azithromycin efficacy of 95% (*10*). We considered up to 10 rounds of TCT and up to 10 rounds of TTT. Treatment rounds would be scheduled, on the basis of WHO guidelines (*9*), at 6-month intervals, with all rounds of TCT being performed before any TTT. Using the Gillespie algorithm (*15*), simulations were run to steady state before starting treatment and then run for an additional 150 months to simulate the time remaining to meet the 2030 deadline. We simulated each scenario 2,240 times and took the mean of the results.

We then considered the effect of population size on the probability of campaign success (i.e., whether a specific treatment campaign would be more successful in areas with greater or fewer persons). We considered population sizes ranging from 100 to 50,000 households, using 2 rounds of TCT followed by 2 rounds of TTT.

Although coverage is likely to be important in determining how successful a mass treatment campaign would be (*16*), recent modeling work has shown that the quality of coverage is also critical (*17*); for example, whether the same persons receive treatment in each round has a substantial effect on the likelihood of EOT. Treatment campaigns that repeatedly miss the same persons are said to have a high level of systematic nontreatment. Models of treatment campaigns usually assume that a random selection of persons receive treatment in each round. However, this likely overestimates the effectiveness of a TCT round. We therefore developed a framework, as laid out in Fitzpatrick et al. (*18*), for mass drug administration, in which we could control person-level treatment correlation between rounds (i.e., if a person is treated in 1 round, how likely are they to be treated in a subsequent round?). For the sake of illustration, we can consider the special cases in which the correlation coefficient (ρ) is 0 or 1. Where ρ = 0, treatment status in 1 treatment round is not associated with the probability of treatment in subsequent treatment rounds, which are independent events. Where ρ = 1, in each treatment round, the same persons are treated, and the same persons are not treated.

We investigated the effect of assigning different values to ρ on the modeled effectiveness of intervention. For ρ>0, we considered treatment status only at household level. So, for ρ = 1, the same households (and everyone in those households) would be treated every round, whereas for 0<ρ<1, each person in the same household has the same probability of receiving treatment, which is different to the persons in other households.

We also considered the use of more frequent, lower-intensity treatment. This could be delivered by, for example, a volunteer offering azithromycin to persons who the volunteer thinks have yaws. We investigated whether this treatment would reduce the required number of rounds of TTT by using an estimated coverage of 5% of infectious persons and their household contacts every month, in addition to rounds of TCT/TTT every 6 months (up to 10 rounds of TCT, and up to 10 rounds of TTT).

## Results

**Figure 3:**
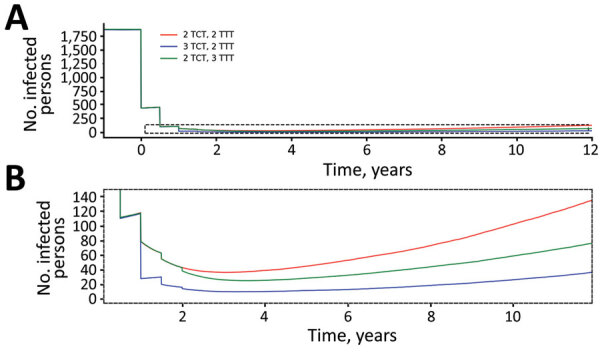
Dynamics of yaws transmission (clinical infectious and latent cases combined, averaged over 1,000 simulations) under 3 different treatment strategies: 2 TCT, 2 TTT (red); 3 TCT, 2 TTT (blue); 2 TCT, 3 TTT (green). A) All parameters tested; B) close-up showing detail of results. Simulations are run to steady state before starting the first round of treatment. Times given are the amount of time (in years) since the first round of treatment. Parameters are inferred from data collected from the Solomon Islands in 2013. TCT, total community treatment; TTT, total targeted treatment.

**Figure 4:**
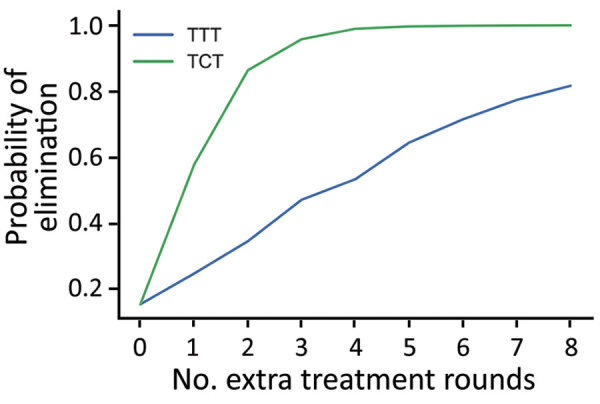
Probability of local elimination of transmission under different intervention strategies consisting of >2 rounds of TCT and >2 rounds of TTT, with varying numbers of additional rounds of TTT (blue) or TCT (green). Each twice-yearly round of TCT has 80% coverage, whereas TTT has 100% coverage and treatment is assumed to have 95% efficacy. All rounds of TCT are performed first before any rounds of TTT begin, which are then also performed twice yearly. Parameters are inferred from data collected from the Solomon Islands in 2013. TCT, total community treatment; TTT, total targeted treatment.

We first consider the dynamics under 3 different treatment strategies, chosen as strategies that have been previously discussed by yaws experts. These strategies, and the probability of EOT calculated for each, are summarized in Table 3. Although an extra round of treatment of either kind is beneficial, an extra round of TCT outperforms an extra round of TTT (Figure 3). In Figure 4, we plot EOT probability for up to 8 additional rounds of TCT or TTT, from a base intervention of 2 rounds of TCT and 2 rounds of TTT. Increasing the number of rounds of TCT increases the probability of EOT more rapidly than including additional rounds of TTT. In fact, an additional 4–5 TTT rounds would be required to achieve the same effect as 1 additional round of TCT.

**Table 3:** Summary of yaws treatment strategies*

Strategy	Rounds of TCT	Rounds of TTT	Probability of elimination
1	2	2	15%
2	3	2	57%
3	2	3	26%

*As shown in Figure 3. TCT, total community treatment; TTT, total targeted treatment.

**Figure 5:**
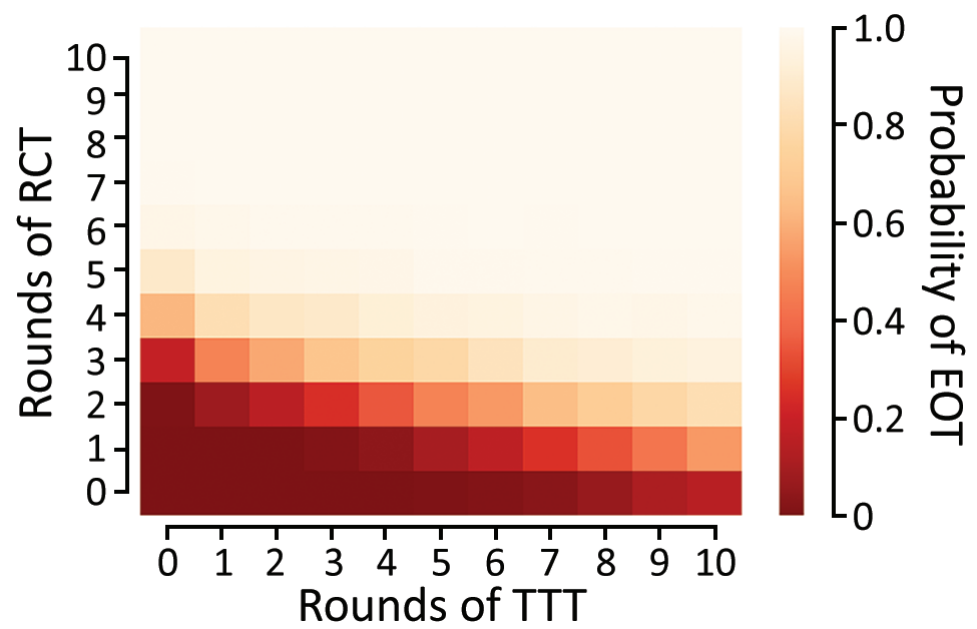
Probability of local elimination of transmission under different intervention strategies with varying numbers of rounds of TCT followed by rounds of TTT treating clinical case-patients and household contacts. Each rectangle in the figure represents a different strategy (consisting of some number of rounds of TCT followed by rounds of TTT). The color of the rectangle shows the probability of elimination of transmission, based on the color bar to the right. Each twice-yearly round of TCT has 80% coverage, whereas TTT has 100% coverage and treatment is assumed to have 95% efficacy. Parameters are inferred from data collected from the Solomon Islands in 2013. TCT, total community treatment; TTT, total targeted treatment.

We extend this further by comparing the effectiveness of 120 different treatment strategies, plus a control strategy in which no antibiotic treatment is provided, in a population of 5,000 households. Any strategy involving <3 rounds of TCT is unlikely to be effective in meeting the 2030 WHO goal for yaws (Figure 5). When we assume a TCT coverage of 90%, we still find that >3 rounds should be considered (Appendix). TTT does not directly precipitate EOT by treating all cases. Instead, multiple rounds of TTT serve to keep infection prevalence low, so that in a small population infection eventually disappears stochastically. Effective population size therefore influences the probability of EOT (i.e., infection in a smaller population is more susceptible to stochasticity). Effective population size refers to the population that interacts, so that an isolated small village will have a small population size, whereas multiple villages sharing schools with substantial between-village intermingling have a larger effective size than the individual villages.

**Figure 6:**
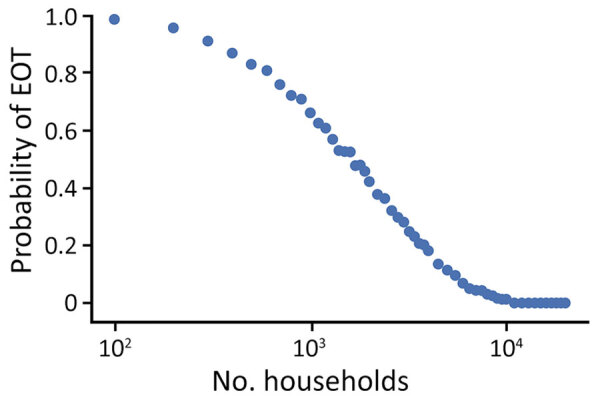
Probability of local elimination of transmission for varying population sizes after 2 rounds of TCT followed by 2 rounds of TTT, 6 months apart. TCT has 80% coverage, and TTT has 100% coverage, and treatment is assumed to have 95% efficacy. Parameters are inferred from data collected from the Solomon Islands in 2013. TCT, total community treatment; TTT, total targeted treatment.

Figure 6 shows EOT probability with changing effective population size using a fixed strategy of 2 rounds of TCT followed by 2 rounds of TTT. As the effective population size increases, the probability of EOT decreases, with the probability approaching 0 at »10,000 households.

**Figure 7:**
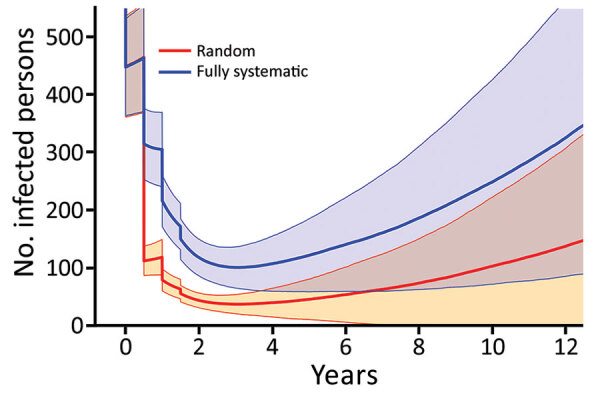
Dynamics of infected yaws (clinical infectious cases and latent cases) under random (red) or fully systematic (blue) coverage when implementing mass drug administration. Simulations are run to steady state before starting the first round of treatment. Times given are the amount of time since the first round of treatment. Treatment involved 2 twice-yearly rounds of TCT, followed by 2 twice-yearly rounds of TTT. TCT has a coverage of 80%, whereas TTT has a coverage of 100% of all infectious persons and their household contacts. Azithromycin efficacy is assumed to be 95%. Shaded regions denote values within 1 SD of the mean value. Parameters are inferred from data collected from the Solomon Islands in 2013. TCT, total community treatment; TTT, total targeted treatment.

**Figure 8:**
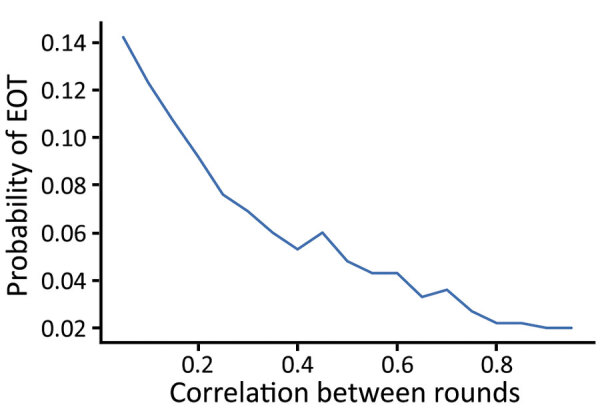
Probability of local elimination of transmission under intervention strategy consisting of 2 rounds of TCT, followed by 2 rounds of TTT treating clinical cases and household contacts as correlation between treatment rounds varies. 0 correlation denotes random treatment, whereas a correlation of 1 denotes fully systematic treatment. Each twice-yearly round of TCT has 80% coverage, whereas TTT has 100% coverage, and treatment is assumed to have 95% efficacy. Parameters are inferred from data collected from the Solomon Islands in 2013. TCT, total community treatment; TTT, total targeted treatment.

As coverage becomes more systematic (so that treatments tend to be given repeatedly to the same persons), the number of cases increases and the probability of EOT decreases substantially (Figure 7). Although random coverage resulted in a probability of EOT of 15%, this result fell to 2% with fully systematic coverage. Each scheme we considered consists of 2 rounds of TCT followed by 2 rounds of TTT. As the correlation between rounds of treatment increases, the probability of EOT decreases substantially, particularly for lower correlations, as we start moving away from random to more systematic treatment (Figure 8).

**Figure 9:**
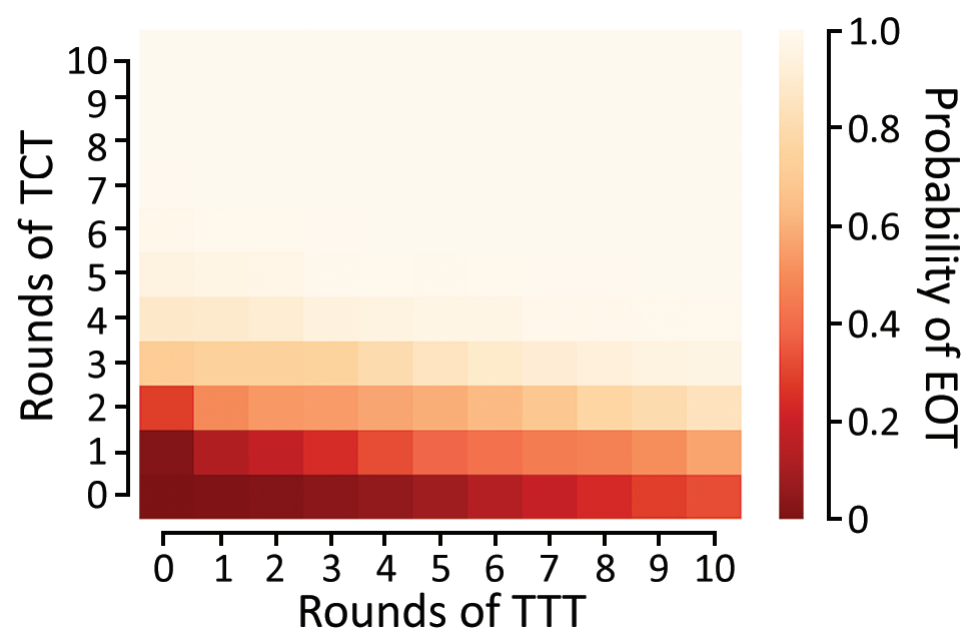
Probability of local elimination of transmission under different intervention strategies with varying numbers of rounds of TCT followed by rounds of TTT treating clinical case-patients and household contacts. Each rectangle in the figure represents a different strategy (consisting of some number of rounds of TCT followed by rounds of TTT). The color of the rectangle shows the probability of elimination of transmission, based on the color bar to the right. Each twice-yearly round of TCT has 80% coverage, whereas TTT has 100% coverage, and treatment is assumed to have 95% efficacy. An additional type of treatment round is administered once a month, giving treatment to 5% of infectious persons and their household contacts. Parameters are inferred from data collected from the Solomon Islands in 2013. TCT, total community treatment; TTT, total targeted treatment.

**Figure 10:**
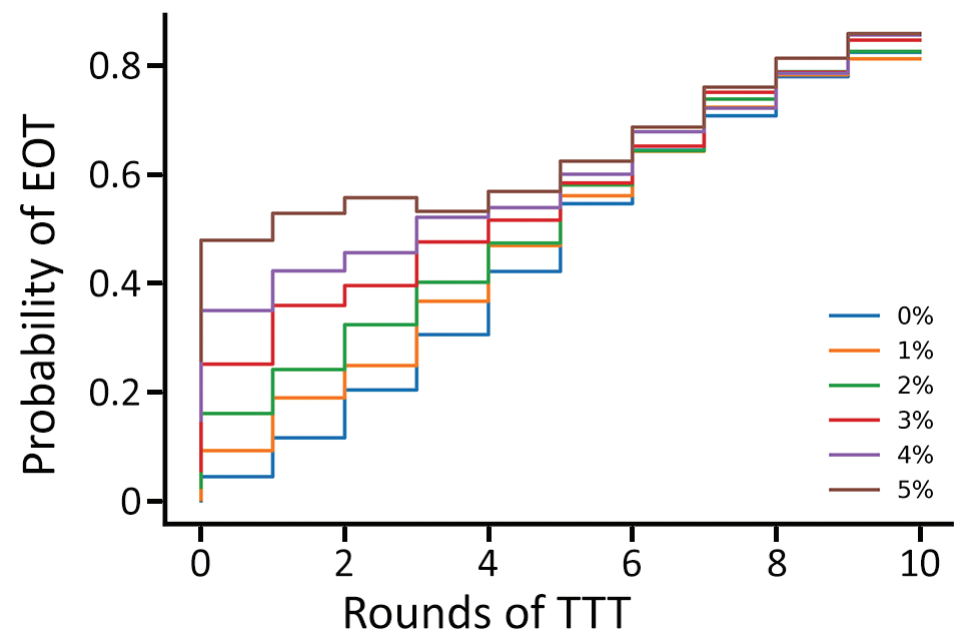
Probability of eradication under a strategy of 2 rounds of TCT with a varying number of rounds of TTT. Additional treatment rounds have coverages of 0% (blue), 1% (yellow), 2% (green), 3% (red), 4% (purple), and 5% (brown). Low-coverage treatment of infected persons and their household contacts occurs once a month. Parameters are inferred from data collected from the Solomon Islands in 2013. TCT, total community treatment; TTT, total targeted treatment.

**Figure 11:**
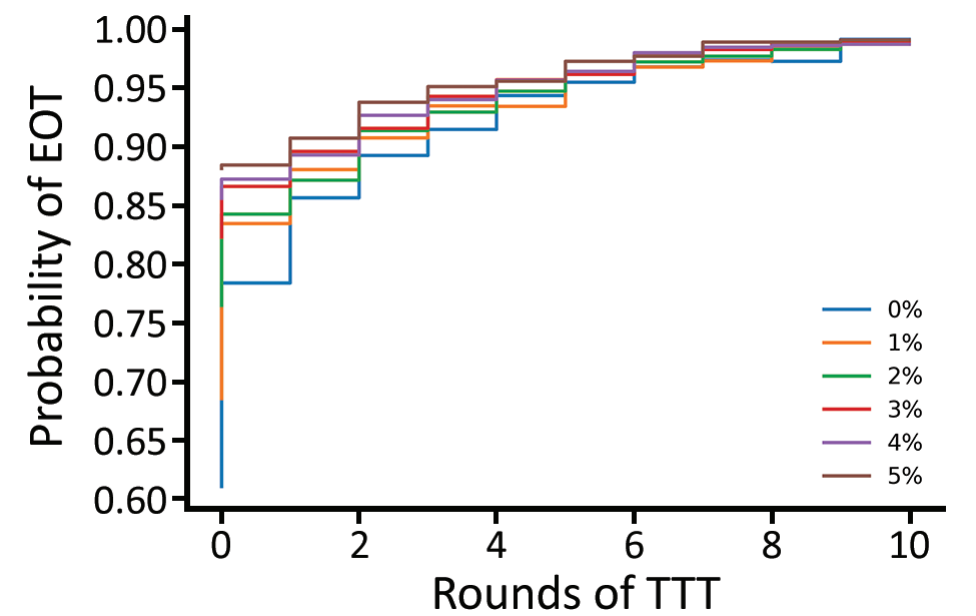
Probability of eradication under a strategy of 4 rounds of TCT with a varying number of rounds of TTT. Additional treatment rounds have coverages of 0% (blue), 1% (yellow), 2% (green), 3% (red), 4% (purple), and 5% (brown). Low-coverage treatment of infected persons and their household contacts occurs once a month. Parameters are inferred from data collected from the Solomon Islands in 2013. TCT, total community treatment; TTT, total targeted treatment.

Since TTT primarily acts by keeping the infectious population at a sufficiently low level that stochasticity eventually leads to elimination, we hypothesize that a lower level of more frequent treatment might act as a potential replacement for TTT. We consider the strategies investigated (Table 3), this time incorporating a monthly volunteer treatment with a coverage of 5% (Table 4). Under a strategy of 2 rounds of TCT followed by 2 rounds of TTT, the probability of elimination increased from 15% to 53% when we incorporated this low-level regular treatment, an increase of 38 percentage points. Similar increases were observed for the other 2 strategies. We noted a very small increase in probability of elimination achieved when performing a third round of TTT, suggesting that TTT has very limited effect with this volunteer treatment. Extending this to the full range of strategies previously considered, we observe that additional rounds of TTT have a reduced effect compared with interventions without background treatment (Figure 9). Further reductions in impact are observed when more rounds of TCT are undertaken (Figures 10, 11). When performing 2 rounds of TCT, increasing volunteer coverage leads to increased probabilities of EOT unless >5 rounds of TTT are undertaken. When using 4 rounds of TCT, increasing the number of rounds of TTT performed results in very little increase in the probability of EOT, regardless of the number of rounds of TTT performed.

**Table 4:** Summary of yaws treatment strategies with and without regular surveillance*

Strategy	Rounds of TCT	Rounds of TTT	Volunteer treatment coverage	Probability of elimination
1a	2	2	0%	15%
1b	2	2	5%	53%
2a	3	2	0%	57%
2b	3	2	5%	71%
3a	2	3	0%	26%
3b	2	3	5%	56%

*Strategies labeled “a’” are those without any regular surveillance, whereas “b” indicates the corresponding strategy with regular surveillance. TCT, total community treatment; TTT, total targeted treatment.

## Discussion

We used a stochastic household-level model of yaws transmission to consider the likely effectiveness of various treatment strategies in the eradication of yaws. As expected, we found that more rounds of TCT and TTT led to higher probability of EOT. However, in our model, EOT was not directly achieved through treatment itself. Rather, TTT served to keep the infection prevalence low, so that yaws eventually disappeared through chance events (i.e., TCT acts to reduce the prevalence of infection to a low level, which is then maintained by TTT until elimination occurs). As such, TCT should be considered the principal driving force for EOT. To efficiently reach elimination, according to our model, multiple rounds of TCT need to be implemented, and TTT should not be considered an effective method for reducing prevalence of infection. Indeed, it would take up to 5 rounds of TTT to achieve the same effect as 1 additional round of TCT. The original Morges strategy, in which only a single round of TCT is advised, is unlikely to enable us to meet the WHO 2030 goal. However, the revised strategy, in which 2–3 rounds of TCT are advised, is more likely to meet this goal if linked to appropriate ongoing surveillance after TCT. However, further rounds of TCT will likely be required if 90% coverage is not attained. This conclusion is consistent with the conclusions of previous modeling work (using the same data) (*14*,*16*), and recent empirical findings from Papua New Guinea (*19*).

Because the effect of chance events was found to be a critical factor in determining success, we next considered the effect of population size on the effectiveness of an eradication campaign. We found that, for a given treatment scheme, the size of the population had a considerable influence on the probability of EOT (i.e., a given strategy is more likely to be successful for smaller than larger populations). The corollary of this finding is that the intervention strategy used in any context should be influenced by the size of the population being treated. This conclusion should be considered in parallel with the conclusion outlined above. For stochasticity to successfully drive EOT, the prevalence of infection after completion of the rounds of TCT needs to be sufficiently small. The larger the population, the greater will be the number of rounds of TCT required to reduce the infection prevalence to the appropriate threshold (Figure 6).

Although the effect of varying treatment coverage levels is widely appreciated, the critical importance of the quality of the coverage is less well understood. The effect of rounds of TCT will be modified by the coverage attained and the level of systematic nontreatment. When we assume treatment with some level of systematic nontreatment, we find that treatment is substantially less effective, given that under these schemes the same persons are treated many times, whereas others are never treated. Because any treatment campaign will likely suffer from some level of systematic nontreatment (in terms of correlation of treatment status between different treatment rounds), programs need to take this into account. As such, when undertaking a treatment campaign, maximizing not just the coverage of the campaign but also the quality of that coverage is beneficial. In short, if we always treat the same persons and miss the same persons, a perpetual reservoir of infection might be maintained, undercutting efforts to interrupt transmission.

Because TTT primarily acts by keeping the infectious population at a sufficiently low level that stochasticity eventually leads to elimination, we hypothesized that a lower level of more frequent treatment might act as a potential replacement for TTT. This end could be accomplished through volunteers handing out azithromycin to infected persons and their household contacts on a monthly (or more frequent) basis. We found this approach to be very effective (theoretically) in increasing the probability of EOT. Once the prevalence of infection was at a sufficiently low level, 5% coverage with ongoing treatment was sufficient to maintain that prevalence until infection was eliminated because of chance. After >4 rounds of TCT, this finding was valid regardless of whether any TTT was performed, perhaps indicating that TTT is redundant. This finding further supports the concept that additional rounds of TCT could be prioritized over TTT, particularly if low-level background antibiotic treatment could be subsequently deployed. Strategies to support ongoing community surveillance deserve further consideration and could link with ongoing regular surveillance for other neglected tropical diseases (*20*,*21*). This approach is supported by a successful elimination campaign in India, in which cash incentives were offered to persons who identified persons with confirmed cases.

Our study has several limitations, which might be addressed in future work. First, we defined a TTT contact as a person in the same household as an infectious person. Extending this to include multiple nearby households, villages, or schools might affect model results. Treating school contacts could be particularly relevant because most new cases of yaws are found in children, which could suggest schools are important settings for transmission. Second, our model includes adults and children in a single class; however, given age-stratified data, we could model treatment effectiveness separately for adults and children, resulting in, for example, age-dependent treatment strategies, such as only treating children, which has been empirically tested for the use of azithromycin in trachoma elimination (*22*). Higher coverage could be more reasonably achieved in younger age groups by yaws programs if this extension of the contact definition was incorporated. Third, spatial heterogeneities might play a role in affecting the transmission of yaws. In parts of the Solomon Islands, persons generally live near the coast and rarely walk through the center of the island. Because an implementation unit is likely to consist of several villages, the population’s spatial distribution and movement patterns might limit the spread of infection. Including this information in our analyses could potentially more closely reflect real-world transmission dynamics.

Reintroduction of yaws from outside the implementation site, although not directly relevant to our research question (which relates to the optimal control strategy within a given area), is possible and should be kept in mind for future yaws modeling work. Similarly, recent reports suggest that nonhuman primates can be reservoirs for yaws bacteria. If further evidence that transmission from nonhuman primates to humans is found, such findings should be considered in future models.

Recent reports have shown that as with the *T. pallidum* subsp. *palldium* bacteria that cause syphilis, the *T. pallidum* subsp. *pertenue* bacteria that cause yaws can develop azithromycin resistance (*19*,*23*). Although penicillin would remain effective in this scenario, such resistance is an important concern for yaws eradication, and how likely implementation strategies are to generate resistance is a critical research question. Although drug resistance is currently a lesser concern than the relapse of latent infection, which is what we investigated in this study, collection of further data on drug resistance should be prioritized so that this possibility can be investigated and incorporated into future models.

In summary, we have shown that the current iteration of the Morges strategy is unlikely to help programs meet the WHO 2030 goal of global yaws eradication. We have suggested alternative strategies that might increase the likelihood of achieving this goal. In particular, we found that further rounds of TCT should be preferred to TTT. We have also shown that population size and quality of coverage can greatly affect the success of a treatment campaign and thus need to be considered in program design. Finally, further consideration should be given to strategies supporting ongoing community surveillance, which could be integrated with ongoing surveillance for other neglected tropical diseases.

Appendix Additional information about modelling treatment strategies to inform yaws eradication.
