# Supplementary material for: Modeling Treatment Strategies to Inform Yaws Eradication
Source: Emerg Infect Dis. 2020 Nov;26(11):2685–93. doi: 10.3201/eid2611.191491 (PMC7588528; doi:10.3201/eid2611.191491)
Supplement: Appendix — Additional information about modelling treatment strategies to inform yaws eradication. [file 19-1491-Techapp-s1.pdf]

Article DOI: https://doi.org/10.3201/eid2611.191491

Modeling Treatment Strategies to Inform Yaws Eradication

Appendix

Finding Alpha

The system can be described at steady state using the following master equation:

$$\frac{\partial P^N_{S,I,A}}{\partial t} = -\left(\left(\epsilon + \frac{\beta I}{N-1}\right) S + \delta(A+I) + \rho A + \lambda I\right) P^N_{S,I,A} + \left(\epsilon + \frac{\beta(I-1)}{N-1}\right)(S+1) P^N_{S+1,I-1,A} \quad (1)$$

$$+ \delta(A+1) P^N{}_{S-1,I,A+1} + \delta(I+1) P^N{}_{S-1,I+1,A} + \rho(A+1) P^N{}_{S,I-1,A+1} + \lambda(I+1) P^N{}_{S,I+1,A-1},$$

subject to the following constraints on S, I and A:

$$S + I + A = N$$

$$S \geq 0, I \geq 0, A \geq 0$$

However, as stated above, this is only applicable once we reach steady state. By applying an intervention, we perturb the system away from steady state and so the same dynamics do not apply. Rather than using a constant, steady state value for ε, we need to consider an ε proportional to the number of infectious individuals in the population. Consider a population of M households, where the h^{th}household is of size $N_h(t)$, with $I_h(t)$infectious individuals at time t(though in our model $N_h$is constant, so will not var.y in time). Then we want to consider an ε of the form

$$\varepsilon(t) = \alpha \frac{\sum_h I_h(t)}{\sum_h N_h(t)}.$$

As ε is a monotonic increasing function of α, there is a unique value of αthat will lead to the same steady state given by each constant ε.

To determine which value of α corresponds to each ε, we would typically just use numerical solvers with the steady state state distribution from the master equation. However, as each household is no longer independent (As each depends on the total number of infectious

individuals in all other houses), equation 1 no longer represents our system. Instead we would have to consider the number of houses in each household state, but this would take too long to manipulate numerically to be of any use. To overcome this, we instead implement a scheme that lets us use the Gillespie algorithm to determine the value of α, but first we need to update the version of the Gillespie algorithm we are using. This will include the update step for ε after each iteration, and will also include the use of convergence diagnostics (Geweke diagnostic) to ensure we are only drawing from the stationary distribution when determining α.

We implement the following scheme to determine the values of α

1. Draw values for ϵ, β, δ *and* $\rho$from their respective posterior distributions.

2. Determine the steady state distribution corresponding to these parameters.

3. Calculate an initial guess for α using $\alpha_0 = \frac{\epsilon_{const}}{\sum_h <I_h>_\infty} \times \sum_h N_h$,where $< I_h >_\infty$denotes the expected number of infectious individuals in household at the endemic steady state.

4. Simulate forward the master equation for each constant ε to approximately determine how long it will take to converge to steady state when using the corresponding α. Do this by finding times t* at which the number of infectious individuals remains within 0.01 of the previously calculated steady states until a predetermined time t_{max}. If it does not converge in this time, keep increasing t_{max}until the master equation converges. We take a burn-in time of $1.5 \times t^*$.

5. Set t_{max}equal to some large period of time (to allow for a sufficiently large number of iterations, and also to ensure convergence), and set the initial condition equal to $\sum_h < I_h >_\infty$.

6. Use a numerical solver such as MATLAB's *fzero* to determine which value of αwill give the same steady state determined previously.

7. Repeat from step 1 until enough samples have been drawn to determine the distribution of α.

Geweke Diagnostic

The Geweke convergence diagnostic (*1*) is a way for us to ensure that the markov chain has converged, so that we only take our time average over samples drawn from the steady state

distribution. It works on the idea that if the mean of the first 10% of iterations is not significantly different from the last 50%, then we can assume convergence occurred in the first 10% of the chain.

Let θ^tdenote the value of the sample drawn at time $t$after an initial burn-in of $n_0$iterations, and let $A = \{t: 1 \le t \le n_A\}, B = \{t: n_B \le t \le n\}$. Then let

$$\underline{\theta}_A = \frac{1}{n_A} \sum_{t \in A} \theta^t, \underline{\theta}_B = \frac{1}{n - n_B + 1} \sum_{t \in B} \theta^t.$$

If the chain has converged at time n_0,then the two means should be equal, and so Geweke's diagnostic should converge to a standard normal as $n \to \infty$. Thus, if the samples from both means are drawn from the stationary distribution, we should have

$$Z_n = \frac{\underline{\theta}_A - \underline{\theta}_B}{\sqrt{\frac{1}{n_A}{\sigma_A}^2 + \frac{1}{n - n_B + 1}{\sigma_B}^2}} \to N(0,1)\ n \to \infty,$$

where ${\sigma_A}^2, {\sigma_B}^2$denote the variances of the two subsamples. This can be used to test the null hypothesis that the two samples are drawn from the same distribution. If they are not, the number of iterations assumed to be burn-in should be increased, and this process repeated. If this fails to work, then it suggests the chains are not converged by the time being considered, and that the chain should be run for longer.

Running Simulations

Simulations were run using computing resources at the University of Warwick. We ran each scenario 10 times on 28 cores on each of 8 nodes. This means that for each scenario, results are determined from a total of 2240 simulations.

Household-level systematic non-compliance

Here we examine a model of systematic non-compliance in which those that have previously attended rounds of treatment are more likely to attend future rounds, and which can be controlled by the magnitude of this correlation between rounds, ρ. We consider this systematic non-compliance at the household level, rather than the individual level. So it is the entire household that either will, or will not attend treatment.

To this end, let $\mathbf{Y_i} = (y_i{}^1, \ldots, y_i{}^N)$, where Ndenotes the population size, and each $y_i \in \{0,1\}$is whether or not members of that particular household attended treatment in round i. Let $\mu_y$denote the coverage we want to achieve. Take$Y_1 = Bernoulli(\mu_y)$, and for subsequent rounds $Y_i = Bernoulli(\lambda_i)$, where

$$\lambda_i = \frac{\mu_y(1-\rho) + \rho \boldsymbol{R_i}}{1 + (i-2)\rho},$$

and $R_i = \sum_{j=1}^{i-1} Y_i$. Then it can be shown (*2*) that $\mathbb{E}[Y_i] = \mu_y, \forall i$ and $corr(y_i{}^k, y_j{}^k) = \rho$. This is actually equivalent to assigning each household a parameter, p_k, that remains fixed for all rounds of treatment that gives their probability of attending a round of treatment

$p_k \sim Beta(\frac{\mu_y(1-\rho)}{\rho}, \frac{(1-\mu_y)(1-\rho)}{\rho})$. In the extreme cases, we can show that the the following models are equivalent:

$$\rho = 0\text{: } Random\ Model$$

Let c denote the desired coverage. In this model, individuals attend treatment with probability c. So in round i, we have $X_i = 1$ with probability c, and 0 otherwise.

Then X_i has mean given by $\mathbb{E}[X_i] = c$, and a variance given by $Var[X_i] = c(1-c)$. We can determine the correlation between rounds as

$$\rho_{X_i X_j} = \frac{cov(X_i, X_j)}{\sigma_{X_i}\sigma_{X_j}} = \frac{\mathbb{E}[X_i X_j] - \mathbb{E}[X_i]\mathbb{E}[X_j]}{c(1-c)} = \frac{c^2 - c^2}{c(1-c)} = 0.$$

$$\rho = 1\text{: } Fully\ Systematic\ Model$$

Let c denote the desired coverage. In this model, one subpopulation of size c will attend every round of treatment, while a sub-population of size (1-c) will never attend treatment. So the correlation is 1 due to each individual doing the same thing every round.

References

1. Geweke J. Evaluating the accuracy of sampling-based approaches to the calculation of posterior moment. In: Bayesian statistics 4. Bernardo JM, Berger JO, Dawid AP, Smith AFM, editors. Oxford: Oxford University Press; 1992.

2. Dyson L, Stolk WA, Farrell SH, Hollingsworth TD. Measuring and modelling the effects of systematic non-adherence to mass drug administration. Epidemics. 2017;18:56–66. PubMed https://doi.org/10.1016/j.epidem.2017.02.002

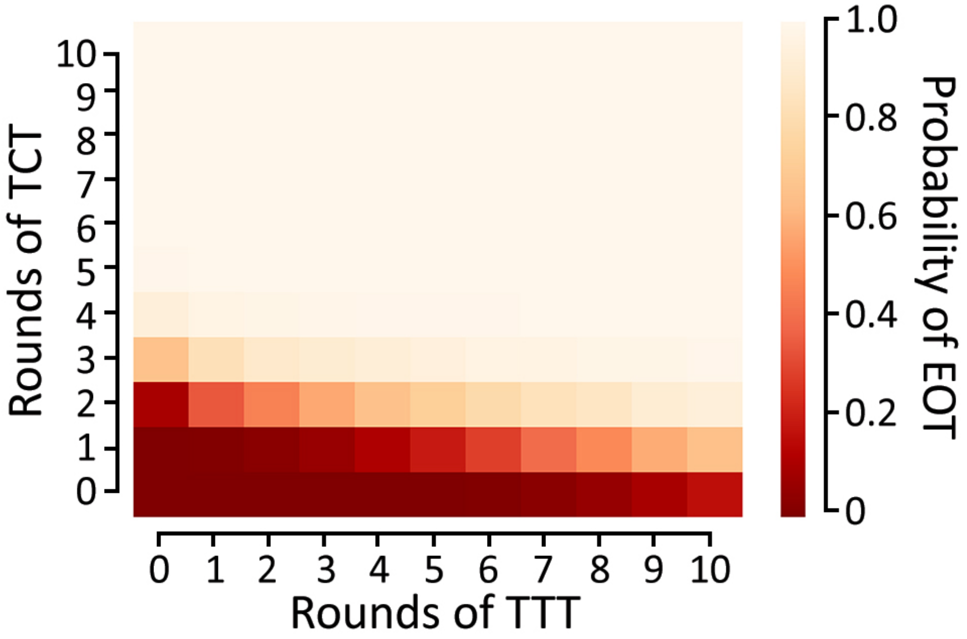

Appendix Figure. Probability of local elimination of transmission under different intervention strategies with varying numbers of rounds of TCT followed by rounds of TTT treating clinical cases and household contacts. Each rectangle in the figure represents a different strategy (consisting of some number of rounds of TCT followed by rounds of TTT). The color of the rectangle shows the probability of EOT, using the color bar to the right. Each twice-yearly round of TCT has 90% coverage, while TTT has 100% coverage and treatment is assumed to have 95% efficacy.
